# Interactions between Humic Substances and Microorganisms and Their Implications for Nature-like Bioremediation Technologies

**DOI:** 10.3390/molecules26092706

**Published:** 2021-05-05

**Authors:** Natalia A. Kulikova, Irina V. Perminova

**Affiliations:** 1 Department of Soil Science, Lomonosov Moscow State University, Leninskiye Gory 1-12, 119991 Moscow, Russia; knat@darvodgeo.ru; 2 Bach Institute of Biochemistry, Fundamentals of Biotechnology Federal Research Center, Russian Academy of Sciences, pr. Leninskiy 33, 119071 Moscow, Russia; 3 Department of Chemistry, Lomonosov Moscow State University, Leninskiye Gory 1-3, 119991 Moscow, Russia

**Keywords:** remediation, biodegradation, lignin-modifying enzymes, extracellular electron shuttles, modification of humic substances

## Abstract

The state of the art of the reported data on interactions between microorganisms and HSs is presented herein. The properties of HSs are discussed in terms of microbial utilization, degradation, and transformation. The data on biologically active individual compounds found in HSs are summarized. Bacteria of the phylum *Proteobacteria* and fungi of the phyla *Basidiomycota* and *Ascomycota* were found to be the main HS degraders, while *Proteobacteria, Actinobacteria*, *Bacteroidetes*, and *Firmicutes* were found to be the predominant phyla in humic-reducing microorganisms (HRMs). Some promising aspects of interactions between microorganisms and HSs are discussed as a feasible basis for nature-like biotechnologies, including the production of enzymes capable of catalyzing the oxidative binding of organic pollutants to HSs, while electron shuttling through the utilization of HSs by HRMs as electron shuttles may be used for the enhancement of organic pollutant biodegradation or lowering bioavailability of some metals. Utilization of HSs by HRMs as terminal electron acceptors may suppress electron transfer to CO_2_, reducing the formation of CH_4_ in temporarily anoxic systems. The data reported so far are mostly related to the use of HSs as redox compounds. HSs are capable of altering the composition of the microbial community, and there are environmental conditions that determine the efficiency of HSs. To facilitate the development of HS-based technologies, complex studies addressing these factors are in demand.

## 1. Introduction

Humic substances (HSs) are ubiquitous in natural and human-made environments such as soil, compost, sewage, natural waters, landfill leachates, and the atmosphere [1,2,3,4]. Consolidated resources of HSs are deposited in sediments, peat, lignites, brown coal, and other organic rocks [5,6]. HSs are the products of postmortal biotic–abiotic transformations of plant, animal, and microbial debris. The formation of HSs occurs by the principle of natural selection [7,8]. This results in the self-organization of supramolecular assemblies of oxidized biomacromolecular precursors such as lignins, polysaccharides, lipids, proteins, and tannins. Extreme structural heterogeneity provides for the relative stability of HSs to biodegradation [8]. As a result, they can be considered as a major natural reservoir of organic carbon and an important sink in both natural and agricultural environments [4]. This fact indicates the essential role of HSs in regulating the global carbon cycle and that the stability and degradability of HSs are a fundamental part of understanding the global CO_2_ budget [9,10]. They play important biospheric functions including transportation, accumulation, regulation, and physiological and protective roles. Therefore, the view regarding the vital role of HSs in maintaining environmental stability is generally accepted [11].

Despite the relative stability of the HSs in response to biodegradation, a number of microorganisms can utilize HSs. The role of microorganisms in the transformation of HSs is crucial for understanding the global carbon cycle [10]. HSs, in turn, beneficially affect the growth of microorganisms. HSs stimulate microbial growth, as a source of nutrients [6,10,12,13,14]. They also play the role of extracellular electron shuttles (EESs), which enables the availability of spatially remote substrates [15,16,17,18]. In addition, HSs increase the solubility of poorly soluble substrates [19,20,21,22]. HSs enhance the survival and growth of microorganisms under unfavorable and adverse conditions due to antioxidant activity [23,24,25]. The beneficial effects of HSs on microorganisms are of particular importance in polluted environments, where the utilization of HSs by microorganisms is closely related to the transformation of both organic pollutants [26,27,28] and inorganic species [29,30,31].

Recently, the potential benefits and research challenges in the agricultural application of humic products were reviewed [32]. The major problems and perspectives related to HS-based technologies were formulated. However, a systematic review of the reported data on the interactions between HSs and microorganisms is still missing. It could lay the grounds for developing the principles of nature-like biotechnologies. In this review, we tried to tackle these needs.

## 2. Humic Substances as a Complex Molecular System

HSs are produced in situ due to chemical, physical, and microbial degradation, as well as (re)polymerization of phenolic and aromatic components such as lignin, tannins, and secondary metabolites [17,33,34]. They represent the dominant form of organic matter (OM) in the environment and are ubiquitous in marine, aquatic, soil, and sedimentary ecosystems, incorporating up to 94% of the total organic carbon [1,3]. HSs are operationally defined according to solubility: the humin fraction is insoluble in the whole pH range, humic acids (HAs) are insoluble at pH < 2, and fulvic acids (FAs) are soluble in the whole pH range. An alkaline-extractable, alcohol-soluble fraction of HA is known as hymatomelanic acid (HMA) [4,7,10].

From trophic and biogeochemical perspectives, HSs are much more refractory to microbial utilization than many other carbon sources [17,35,36]. This is reflected in the large ^14^C age residence time of HSs in soil, which varies from 250 to 3000 years [7]. For freshwater (riverine) HSs, the reported values of carbon isotopic ratio (Δ^14^C) ranged from −44 to −247‰ [37], giving a residence time of 360–2280 years. The stability of HSs is determined by molecular recalcitrance, as they are comprised of an extremely heterogeneous complex mixture of molecules [4,8,38,39,40]. The high complexity of the molecular organization of HSs is reflected in the extreme diversity of the molecular space of HSs: it was found that CHO formulae identified in freshwater FAs by electrospray ionization Fourier transform ion cyclotron resonance mass spectrometry (FTICR MS) covered up to 80% of all possible CHO combinations with molecular weights in the range of 250–650 Da [39].

Extreme molecular diversity renders the assignment of an exact chemical structure to HSs impossible [39,41]. There are different views with regard to the molecular organization of HSs. One of them sees HSs as aggregates of low-molecular-weight compounds [41,42,43]. The debate on the structure of HSs is not over yet [40,44,45]. It is generally accepted that HSs are composed mainly of aromatic, aliphatic, phenolic, quinonic, and N-derived components, which are covalently bound through C–C, C–O–C, and N–C bonds. HSs have an abundance of oxygen-containing functional groups (carboxyl, phenol, alcoholic ketone, ester, and ether), which dominate their properties and structure [10,46].

Both hydrophilic and hydrophobic fragments can be found in HSs, rendering them surface-active [47]. The heterogeneity of functional groups results in the high reactivity of HSs towards organic and inorganic pollutants [48,49,50]. Surface activity determines solubilization phenomena in the presence of HSs with regard to poorly soluble hydrophobic organic compounds [19,21,51]. Binding to HSs decreases the bioavailability of toxicants [52].

The HSs can bind not only toxicants but also nutrients [4,46,53]. Nitrogen (N) can be incorporated into HSs both in the form of neutral and protonated NH_2_ groups in amino acids and sugars, as well as NH_4_^+^ [54]. Soil HSs contain up to 10% P in the form of inorganic phosphate species, with phosphate monoesters as the dominant species [55]. The bioavailability of metals in soils is due to complexation with acidic groups of HSs [4]. This is why the metals bound to HSs are readily bioavailable to microorganisms [56]. The reported values of the content of Ca, Mg, Fe, Mn, and Zn in soil HSs are in the ranges of 0.3–5.6 mg/g, 0.1–0.4 mg/g, 0.3–2.2 mg/g, 0.1–1.3 mg/g, and 0.02–0.3 mg/g, respectively [57]. Prerequisites for the susceptibility of humic acids to microbial utilization and transformation with various metal contents were summarized in [46]. The peculiar properties of HSs described above indicate that they can be a source of nutrients for microorganisms.

HSs are rich in phenoxyl radicals, which are capable of binding a variety of organic and inorganic molecules and elements including amino acids, peptides, sugars, and lignin fragments [44]. Vast data sets are reported on the presence of biologically active molecules in HSs (Table 1), including amino acids, carbohydrates, and lipids [33,38,53,58,59]. Along with the compounds listed in Table 1, many other biologically active substances have been detected in humic materials, such as melanin [10]; phenolic acids [53]; quinone moieties [17], triterpenoids [33]; amino sugars [54]; and pyrrole, pyridine, or pyrazine [60].

A part of N-bearing moieties of HSs, mainly amino acids, seems to be readily available for microbial utilization due to the hydrolytic cleavage of macromolecules by extracellular enzymes [53,58,73]. The cleavage of peptidic bonds can be successfully performed by bacterial aminopeptidase (e.g., cleavage of amino acids from N-terminal polypeptide chains) [74,75]. Bacterial consumption of carbohydrates was not dependent on whether they were bound to HSs or not: as much as 70% of carbohydrates consumed could be associated with HSs [58]. The reversible incorporation of lipids into HSs and an opportunity of microbial reworking have been also demonstrated [69]. The data above indicate that biologically active compounds incorporated into HSs might be readily bioavailable. At the same time, some incorporated moieties become more resistant to biodegradation because of several protection mechanisms including covalent binding and physicochemical encapsulation within the hydrophobic core of the aromatic skeleton and formation of organo-mineral complexes [40].

A gradual trend over geologic times toward a depletion of carbohydrates and amino acids along with a dominant accumulation of aromatic compounds is well known from the temporal sequence of coalification stages: plants–composts–peats–lignite–hard coals [4,76]. As a result, HSs possess an aromatic backbone containing phenolic and quinonic units [38], which accounts for up to 30–60% [44]. The presence of phenolic and quinonic fragments in HSs determines their redox activity. Phenolic moieties were suggested as major electron-donating structures, whereas quinones are considered to be one of the principal acceptor moieties present in HSs [77,78]. Phenolic moieties mainly include mono- and polyhydroxylated benzene units and have antioxidant properties [77,79]. By quenching reactive oxidants, phenolic moieties protect functional groups in HSs from oxidation and therefore play an important role in their stability in the environment [79,80]. From the point of view of the interaction with microorganisms, antioxidant activity of HSs determines the capability of HSs to mitigate the inhibition of microbial growth under adverse environmental conditions through interrupting radical reactions and preventing damage to cell membranes and biological macromolecules [81,82]. The mitigating activity of HSs has been repeatedly demonstrated for all groups of microorganisms, including bacteria and fungi [24,81,83,84,85].

Quinonic moieties, in turn, were identified as the main functional groups conferring the electron transferring capability to HSs [86,87,88]. They enable the extracellular electron shuttling properties of HSs, providing microbes with access to the remote substrates [18]. The presence of quinonoic moieties makes it possible for HSs to serve as terminal electron acceptors [26,89,90]. Recent reports suggest that other functional groups, such as nitrogen and sulfur functional groups, could also contribute to the electron transferring capability of HSs [88,91]. The reported redox potential of HSs is in the range from +0.1 to +0.6 V, so HSs can serve both as acceptors and as donors of electrons [92].

Overall, a brief review of the existing data demonstrates that HSs can serve as a source of nutrients or biologically active compounds. Another important role of HSs is electron shuttling, which is heavily utilized in microbial redox reactions. In addition, the effect of HSs on microorganism functioning can be related to the ability of HSs to increase or decrease the bioavailability of pollutants, including toxicants.

## 3. Utilization, Degradation, and Transformation of HSs by Bacteria

A large variety of bacterial consortia capable of degrading HSs were isolated from soil [9,13,14,93,94], coal [20], or aquatic environments [2,95,96,97,98], including marine and estuarine waters [6,99,100]. According to the data presented by Yanagi and coauthors [14], the population of total bacteria that were capable of degrading soil HAs varied from 0.1 × 10^6^ to 2.8 × 10^6^ CFU g^−1^ of soil, forming 0.2–3.5% of the total microbe density.

### 3.1. Bacteria Capable of HS Degradation and Utilization

The ability of individual bacterial isolates to degrade HSs was firstly reported in the beginning of the 1960s when Mishustin and Nikitin published a study on microbial degradation of HSs related to several soils and demonstrated that bacteria of the genus *Pseudomonas* were most active in humate decomposition [12]. Later, the degradation potential towards HSs was also demonstrated for the bacteria belonging to the genera *Bacillus* [101,102,103,104], *Agrobacterium* [101], *Clostridium* [105], and others. Hutalle-Schmelzer and Grossart [106] found that the addition of HSs favored the growth of *Betaproteobacteria* (namely *Polynucleobacter*, *Acidovorax*, *Herbaspirillum*, and *Methylophilus* species). Recently, several isolates of *Alphaproteobacteria* and *Gammaproteobacteria* were found to grow on media containing HSs as the sole carbon source [6,107]. Some genera of HS-degrading bacteria are presented in Table 2.

The large variety of bacteria capable of degrading HSs and their occurrence in soil and natural water indicate that bacterial decomposition of HSs is a widespread phenomenon. HS-degrading bacteria can be mostly affiliated with the Gram-negative *Proteobacteria* (Table 2) with an outer membrane containing lipopolysaccharides. Among *Proteobacteria,* the class *Alphaproteobacteria* can be mainly found [6,100,101,107]. These bacteria can grow at very low levels of nutrients and have unusual morphologies, such as stalks and buds [118]. *Betaproteobacteria* is another class of humic-degrading bacteria [106,107,108]. It often uses nutrients that diffuse from areas of anaerobic decomposition of organic matter (hydrogen gas, ammonia, methane) and includes chemoautotrophs. Still, bacteria capable of HS utilization cannot be unambiguously assigned to a certain taxonomy group.

As can be seen from Table 2, many of these bacteria are Gram-positive and belong to other phyla than *Proteobacteria*, namely *Actinobacteria* or *Firmicutes*, or Gram-negative *Bacteroidetes*. This diversity is not surprising given the structural complexity of HSs. In addition, Rocker and coauthors [6] demonstrated that the taxonomic affiliation of bacteria capable of the utilization of HSs as a sole carbon source depended heavily on the bacterial inoculum source. In the experiment with the marine inoculum, a total of 19 single isolates or isolate groups were obtained, and the majority of isolates (74%) and 11 isolate groups (58%) were affiliated with *Alphaproteobacteria*. On the other hand, when the estuarine inoculum was used, the largest fraction of isolates (46%) was affiliated with *Gammaproteobacteria*.

The reported data allow a conclusion to be drawn that HS degradation in the environment seems to be a complex multistep process rather than decomposition by individual bacteria strains. This can be also supported by the distinct changes in the bacterial community during incubation in the presence of humic materials [6,100,106] and by the observations that not a single isolated strain is capable of HS degradation to the same extent as the natural bacterial community [100]. Hutalle-Schmelzer and Grossart [106] found that humic material addition to the bacterial plankton communities resulted in a decline in the number of bacteria related to *Actinobacteria*, whereas the preferential growth was observed for specific *Betaproteobacteria* populations in the presence of HSs. In addition, an *Alphaproteobacterium* (related to *Roseisalinus* group), several *Betaproteobacteria* bacteria (related to *Herbaspirillum*, *Acidovorax*, *Comamonas*, and *Anaeromyxobacter* groups), a *Deltaproteobacterium* (related to the *Anaeromyxobacter* group), and a *Bacteroidetes* (related to the *Brumimicrobium* group) were not detected in the initial pattern but were detected only after HS addition. Rocker and coauthors also found several isolates of *Actinobacteria* that were able to grow on the media containing HSs as the sole carbon source [6].

Given that microorganisms cannot take up large molecules directly due to the biological membrane barrier, pinocytosis and phagocytosis are usually assumed to be responsible for humic material uptake [119]. The experiments were conducted using dual-labeled humic-like substances (^15^N, ^13^C) based on the assumption that if pino- or phagocytosis were used to take up the HSs, both ^15^N and ^13^C would be taken up in the same stoichiometric ratio as they appear in the labeled material. However, in experiments with three coastal phytoplankton strains known to utilize HSs, *Synechococcus* sp., *Amphidinium carterae*, and *Thalassiosira* cf. *miniscula*, no significant uptake of ^13^C was measured, indicating that the HSs were not taken up whole using these mechanisms [119]. Nevertheless, the capability of bacteria in uptaking tritium-labeled HSs was demonstrated using tritium-labeled humic materials. The measured amounts of HSs found in the cell interior ranged from 23 to 167 mg kg^−1^, accounting for about 20% of total HS uptake by the cells in the case of HAs and reaching 100% in the case of FAs [120]. The capability of bacteria to degrade humic materials is usually related to enzymes excreted by bacteria, and extracellular enzymatic degradation is supposed to be the first step required for the bacterial uptake of humic materials [121].

### 3.2. Extracellular Aerobic Degradation of HSs

As early as 1991, Crawford and Gupta [109] described a non-oxidative enzyme lignite depolymerase that was proposed to be involved in the depolymerization of HAs obtained from weathered lignite by several Gram-positive and Gram-negative bacteria. Bronk and coauthors [122] underlined the role of extracellular proteolytic enzymes that are able to break down large polymer HSs into their smaller constituent molecules, which can then be taken up by the cells. To elucidate the potential mechanism of leonardite degradation/liquefaction by the alkali-producing bacterial community, the activities of ligninolytic enzymes (Mn-peroxidase, ligninperoxidase, and laccase) and esterase were measured during the degradation [20]. Mn-peroxidase activity was found to be activated by leonardite, whereas esterase activity was not affected. It should be also noted that the increase in the pH of the media during coal decomposition evidenced a non-enzymatic pathway of degradation. Carlsson and coauthors demonstrated that bacterial aminopeptidase and P-glucosidase activity was stimulated by the addition of riverine HSs and that bacteria also utilized the released amino acids from the humic material [53]. The use of cell-surface enzymes capable of cleaving amino groups is a potential mechanism used to access the approximately 50% of humic-derived N that is in the form of amino acids, amino sugars, NH_4_^+^, and nucleic acid bases [123]. Kontchou and Blondeau [103] detected peroxidase activity in culture filtrates of *Streptomyces viridosporus* growing in a medium containing glucose and mineral salts. Byzov and coauthors [107] explained the ability of bacteria to decompose HSs by means of extracellular polyphenol oxidase activity. Other scientists [124] related HS bacterial degradation to cellulases, endohemicellulases, and debranching and oligosaccharide-degrading enzymes.

Actinobacteria are supposed to be able both to produce and to degrade HSs [9]. To date, about 10 genera of *Actinobacteria* have been demonstrated to be effective humus degraders (Table 2). Most of the actinobacteria isolates were determined to be *Streptomyces* spp., and they apparently grew at the expense of carbohydrates, amino acids, and other easily decomposable structural units of humic materials [9]. Some researchers found that actinobacteria could not use HSs as the sole source of C [103,115] and that glucose should be added to the culturing media. However, many isolates of actinobacteria (species of *Dactylosporangium*, *Micromonospora*, *Microtetraspora*, *Nocardia*, *Streptomyces*, *Streptosporangium*, and *Thermomonospora*) were found to grow in the media containing HSs as the sole C and N source [113].

### 3.3. Anaerobic Transformations of HSs

Unlike enzymatic processes involved mainly in the aerobic degradation of HSs, less well understood are the anaerobic pathways [105,124]. A role of the anaerobic ammonium oxidation (Anammox) process in HS degradation under anaerobic conditions was shown for the heterotrophic bacteria consortium by [2]. The bacteria capable of utilizing HSs as the sole carbon source in this process were isolated from the consortium and identified as four facultative anaerobic strains, namely *Bacillus* sp., *Paenibacillus* sp., *Bacteroides* sp., and *Staphylococcus* sp. In the consortium, *Bacillus* dominated with 45%, followed by *Paenibacillus* with 25%, and *Staphylococcus* occupied a relatively low content with 13%. The authors found the correlation between anaerobic oxidization of HSs and sulfate reduction: HSs served as electron donors under those conditions. It was hypothesized that the oxygen functional groups, such as C=O of quinoid and ketones, C–O of carboxylic acids, and phenolic O–H, might be of particular importance here. Recently, the electron-donating properties of HSs were also demonstrated in the process of dissimilatory iron reduction in the presence of an *Ignavibacterium/Melioribacter* [124].

It should be underlined, however, that along with HS oxidation under anaerobic conditions, more often HSs serve as terminal electron acceptors supporting microbial metabolism rather than electron donors [15,125]. Many microorganisms found in soils and sediments are able to use HSs as an electron acceptor for the anaerobic oxidation of organic compounds and hydrogen. This electron transport yields energy to support their growth. The utilization of HSs as electron acceptors will be discussed later.

### 3.4. HS Transformation in the Gut of Soil Macro- and Microfauna

Bacterial degradation of HSs determines to a significant extent the transformation and stability of HSs with regard to the soil fauna [40]. The feeding activity of the soil macrofauna, particularly earthworms and humivorous insects, can markedly change the physicochemical properties of soil organic matter and, therefore, plays a key role in its turnover. The intestinal processes in the gut of humivorous insects have been investigated primarily with soil-feeding termites [126]. The true soil-feeders (feeding group IV; [127]) ingest mineral soil and are able to utilize the most recalcitrant soil components. They comprise the *Cubitermes* branch of the *Termitinae*, an important component of the soil macrofauna in African tropical forests and wet savannahs [128]. According to [129], about half of the 2200 termite species now referenced thrive on the humic compounds of the soil and contribute to the soil humification process. Estimations of the annual soil consumption by *Cubitermes* species range from 1.2 to 4.5 kg m^2^ [130]. The gut of soil-feeding termites is highly compartmentalized and characterized by an increase in the length and volume of the paunch, allowing a sequential transit of long duration, up to 48 h [131]. The use of microsensors in different gut sections has clearly demonstrated that during this transit, the organic matter is submitted to different physical and chemical environments, mostly due to pH and oxygen and hydrogen pressure variations [132,133,134]. The high alkaline level of the paunch (up to pH 12) is the most important and appears to be a general feature for most of the major hindgut compartments of soil-feeding termites [135]. Another important feature of the gut of soil-feeding termite is the presence of a high density of bacteria, reaching 10^8^ to 10^9^ cells per mL of gut [136]. The bacterial community is characterized by a high level of active Archaea methanogen microorganisms and a relatively low density of carbohydrate-fermenting bacteria [135].

Studies with ^14^C-labeled humic model compounds demonstrated the capacity of *Cubitermes* species to mineralize HSs [130,137,138]. Soil-feeding termites were hypothesized to exploit the peptidic component of HSs as a dietary resource, and amino acids seemed to be important substrates for their intestinal tract microbiota. There is convincing proof that soil-feeding *Termitinae* are able to mobilize and digest the peptidic components of soil HSs [130,137]. The alkaline hydrolysis of the humic compound ingested could lead to the liberation of a large part of protein nitrogen, which could further be degraded by the gut microflora [135]. In addition, the alkaline digestion systems may dissociate the humic–mineral complexes and enhance the solubility of humic compounds [139]. The extreme gut alkalinity in the anterior hindgut and alkali-stable and HA-tolerant proteinases were proposed to play a key role in this process [130]. It has been shown that Fe(III) in the soil ingested by soil-feeding termites could be almost completely reduced within their intestinal tract [140]. Assuming the redox activity of HSs, the latter was probably related to the microbial reduction of Fe(III) by bacteria in the digestion systems of soil-feeding termites under a lack of oxygen.

Another important group of humivorous soil macrofauna is the larvae of cetoniid scarab beetles, which resemble the termite gut with respect to strong midgut alkalinity, high concentrations of microbial fermentation products, and the presence of a diverse microbial community [141]. The larva of *Pachnoda ephippiata* was demonstrated to be able to digest the humic acid stabilized residues of polysaccharides, peptides, and cellulose, whose hydrolysis products form the substrates of the intestinal microorganisms [142]. Hobbie and coworkers [143] showed that the reduction of Fe(III) and HSs also takes place in the alkaline guts of scarab beetle larvae *Pachnoda ephippiata*. The authors demonstrated that sterilized gut homogenates of *Pachnoda ephippiata* were not able to convert Fe(III) to Fe(II), indicating an essential role of the gut microbiota in this process. From Fe(III)-reducing enrichment cultures inoculated with gut homogenates, they isolated several facultatively anaerobic, alkali-tolerant bacteria that were closely related to metal-reducing isolates in the *Bacillus thioparans* group. The rate of dissimilatory Fe(III) reduction by the bacterial isolate was strongly stimulated by the addition of the redox mediator 2,6-antraquinone disulfonate and by redox-active components in the fulvic acid fraction of humus. The authors concluded that the lack of oxygen and the solubilization of HSs in the extremely alkaline guts of humivorous soil fauna provide favorable conditions for the efficient reduction of Fe(III) and HSs by a primarily fermentative microbiota [143].

Similar to the case with soil-feeding termites, the humivorous larva of the scarabaeid beetle *Pachnoda ephippiata* is able to use HSs as a source of nitrogen [142]. A study of the transformation and mineralization of synthetic HAs by the larva of *Pachnoda ephippiata* demonstrated that HAs were solubilized in the alkaline midgut of the larva, resulting in the release of amino acids and NH_4_^+^ formation due to their further mineralization [142]. The authors concluded that not only microbial biomass but also the nitrogen-rich components of HSs are important dietary components for humivorous insects and that the feeding activities of soil macroinvertebrates strongly affect the transformation and mineralization of soil organic matter. Along with nitrogen, the transformation of HSs by the larvae of the scarabaeid beetle *Pachnoda ephippiata* led to an increase in the levels of available P due to high alkaline phosphatase activity in the alkaline midgut of the larvae [144]. This study suggests that the feeding activities of humivorous larvae would affect the amount of soil P available to plant growth.

In contrast to the soil-feeding termites and scarabaeid beetle larvae, geophagous earthworms, which also can transform HSs and are the dominant soil fauna in the temperate and tropical ecosystems [145], do not possess an alkaline digestion system [40]. The capability of two geophagous earthworms, *Metaphire guillelmi* and *Amynthas corrugatus*, to digest the proteinaceous component of HSs was demonstrated by Shan et al. [40]. HS model compounds were specifically ^14^C-labeled either for the aromatic or the proteinaceous component and were then added to soil incubated with the geophagous earthworm species. The mineralization rate of the proteinaceous component of HSs was 1.4–2.0-fold higher in the presence of earthworms as compared to the soil without earthworms, whereas the mineralization rate of the aromatic component was slightly lower (1.2-fold), stimulated only by *A. corrugatus*. The stimulated mineralization was accompanied by a transformation of radiolabeled HSs due to the selective digestion and assimilation of the peptidic component of HSs by means of an incorporation of radiolabeling into the earthworm tissues. The gut proteases were proposed to contribute to a great extent to the selective digestion and mineralization of the peptidic component of HSs. Mineralization of the aromatic component of HSs, in turn, referred to the gut peroxidase activity, which was higher in endogeic *A. corrugatus* than in the anecic *M. guillelmi*. Digestion of the proteinaceous component of HSs was found to show that recalcitrant HSs may be one of the nutrient sources of geophagous earthworms. Recently, Byzov and coauthors [107] demonstrated the participation of *Aporrectodea caliginosa* and *Eisenia fetida* in the decomposition of coal HAs. Overall, 59 intestinal bacteria out of 81 were capable of growing when HSs were a sole carbon source. The authors concluded that polyphenol oxidases excreted by bacteria play a crucial role in HS degradation.

## 4. Utilization, Degradation, and Transformation of HSs by Fungi in Soil

### 4.1. Fungi as HS Degraders

Although bacteria dominate in the environment and participate in the transformation of HSs, their ability to degrade stable macromolecules such as HSs is limited [9,10,100,146]. Fungi are more efficient humic substance degraders [10]. Fungi, which are active in the decomposition process, mainly include ascomycetes and basidiomycetes (Table 3), which are common in the upper layer of forest and grassland soils [10]. Estimates performed by Yanagi and coworkers showed that the density of HA-degrading fungi in soil ranged from 1.9 × 10^4^ to 14.9 × 10^4^ CFU g^−1^ soil, or 2.4–12.5% of total fungi density [14].

The degrading activity of fungi is supposed to be closely related to the lignin-modifying enzymes (LMEs). These are lignin peroxidases (LiPs), manganese-dependent peroxidases (MnPs), versatile peroxidase (VP), and laccase (Lac). LMEs can oxidize phenolic compounds, thereby creating phenoxy and carboxy radicals, while nonphenolic compounds are oxidized via cation radicals. The resulting unstable radicals can then undergo either condensation and polymerization or further degradation, and even mineralization [10]. LiPs and MnPs oxidize nonphenolic aromatic compounds with high oxidation–reduction potentials, while laccase oxidizes nonphenolic aromatic compounds with relatively low oxidation–reduction potentials. In the presence of low-molecular-weight mediators, laccases can also oxidize nonphenolic substrates with high oxidation–reduction potentials [167]. Due to the unique ability of nonspecific oxidizing enzymes to react with a variety of aromatic substrates, white rot fungi, which are the most active producers of LMEs, have been found to be the most efficient degraders of HSs [10]. In some cases, the role of enzymes other than LMEs was also demonstrated. Along with the enhanced activity of peroxidase and phenoloxidase enzymes, coal biosolubilization by two fungal strains, *Trichoderma* sp. and *Penicillium* sp., was accompanied by increased activity of extracellular esterase [154]. Detailed reviews of HS degradation by fungi and the role of LMEs in it can be found elsewhere [9,10].

Especially interesting is the fact that only fungi affiliated with the phyla *Basidiomycota* and *Ascomycota*, i.e., so-called “higher fungi” representing the subkingdom Dikarya, were found to degrade humic material. Though the presence of fungi belonging to other phyla than *Basidiomycota* and *Ascomycota* in the soil organic horizons, such as *Blastocladiomycota*, *Glomeromycota*, *Mucoromycotina*, *Chytridiomycota*, *Neocallimastigomycota*, and *Zygomycota*, has been evidenced [168,169,170], not a single isolate capable of transforming HSs has yet been determined from the above-listed phyla. The oxidative activity of some fungi of the arbuscular mycorrhizal (AM) hyphae-associated microbes (mainly *Glomeromycota*), resulting in an oxidative polymerization humic, was proposed [171]. AM-induced transformation of HSs is unlikely to fully revert the catabolic processes leading to the release of mineral nutrients and energy bound in the soil organic matter, but it is definitely a subject worthy of further attention, first of all from a carbon sequestration point of view [172]. Very detailed reviews of the degradation of HSs by fungi can be found in [10] and [173].

### 4.2. Structural Alteration of HSs Caused by Fungal Utilization

Bacterial and fungal utilization of HSs results in the alteration of HS properties, including a decrease in molecular weight [96,147,148,154,155,174], a loss of carbohydrates [13,175], a loss of aliphatics and an increase in aromatics [13,38,73,96,176,177,178,179], increased aromaticity [147], utilization of the polysaccharides [180,181], a loss of peptides [40,180], alteration of the C/N ratio [9,13,40,54], and oxidation [96]. Peroxidase and phenoloxidase enzymes, which are excreted by microorganisms to utilize HSs, can catalyze not only oxidative polymerization of phenolic moieties of HSs but also the oxidative coupling of phenol and aniline pollutants [182]. This makes the use of the processes of transformation of HSs by microorganisms a promising basis for the development of new products based on HSs or on the HS-containing organic rocks. On the other hand, the ability of HSs to mitigate the negative effect of adverse environmental factors on microorganisms allows us to consider HSs as compounds that can expand the application of microorganisms in bioremediation technologies.

## 5. HSs as Mediators of Microbial Redox Reactions

### 5.1. Reduction of HSs by Microorganisms

HSs are redox-active due to their highly condensed aromatic structures rich in quinone/hydroquinone moieties. HSs have been demonstrated to have three distinct roles as electron carriers, namely (1) electron acceptors for respiration, (2) redox mediators for reduction processes, and (3) electron donors to microorganisms [17,183,184]. HSs act as electron acceptors and electron mediators for microbial respiration and the oxidation of several substances, including organic compounds [15,16,185,186,187], hydrogen [15], and metals [86,124,188]. HSs themselves can act as terminal electron acceptors in anaerobic microbial respiration [189]. As electron donors, HSs could aid microbial respiration and the reduction of nitrate [190], sulfide [191], and Fe(III) oxides [17,52,124,192,193,194]. Though all three types of electron transfer can be executed by HSs, under anoxic conditions HSs are most often considered as terminal electron acceptors during microbial respiration and as electron shuttles driving the redox bioconversion of metals and organic molecules [15,16,17,185,187,195].

Initially, the ability of HSs to act as terminal electron acceptors was observed in the experiments evaluating humus as a chelator of Fe(III) to improve the iron solubility and hence the benzene biodegradation by a Fe(III)-respiring consortium in sediments [16]. It was demonstrated that the high stimulation of biodegradation was due to HSs acting as direct electron acceptors for anoxic benzene degradation rather than chelating iron [15]. HSs reduced by microorganisms can further transfer electrons to insoluble minerals or refractory organic pollutants [186]. Therefore, there has been a growing interest both in humic-reducing microorganisms (HRMs) and the reduction of extracellular substrates mediated by HSs in recent years [124,186]. Nowadays, it is well recognized that HRMs are widespread in nature (Table 4).

HRMs can be found in different environments, mainly with circumneutral pH [193]. Reported estimated population densities range from 10^1^ to 10^6^ cells per g of water-saturated sediment or water, and the most probable numbers (MPNs) of HRMs are seemingly always at least equal to the numbers of Fe(III)-reducers [210]. Along with Fe(III)-reducing microorganisms, HRMs were also found among sulfate-reducing, methanogenic [186,211], and fermenting [212] bacteria. The absence of HRMs has so far only been described for an acidophilic Fe(III)-reducing microorganisms [213], which most probably relates to the higher solubility of Fe(III) at acidic pH when HSs are presented as a solid phase [210].

More than a hundred HRMs belonging to the genera *Desulfitbacterium*, *Geobacter*, *Bacillus*, *Shewanella*, and many others have been reported [186,196]. *Proteobacteria, Actinobacteria, Bacteroidetes*, and *Firmicutes* are the predominant phyla of HRMs (Table 4). Microbial reduction of HSs enhances the capacity of microorganisms for reducing less accessible electron acceptors, because HSs can serve as extracellular electron shuttles (EESs) between the HRM and the substrate [18]. EESs in their reduced state transfer electrons to a distant extracellular oxidant, and then EESs can return to the cell in the oxidized state, whereupon they are re-reduced. It is the cycling of EESs and their facilitation of electron transfer without the cell that underpins their important physiological functions [18]. Reduced HSs can be oxidized directly by metal oxides naturally present in soils and sediments, such as Fe(III) and Mn(IV) [17].

### 5.2. Electron Shuttling

Bacterial species can use HSs as electron shuttles or terminal electron acceptors to support anaerobic oxidation of organic compounds, such as acetate, formate, ethanol, pyruvate, lactate, propionate, and others. Alternatively, the reduction of HSs was also observed by fermentative bacteria *Propionibacterium freudenreichii*, *Enterococcus cecorum,* and *Lactococcus lactis* during the oxidation of glucose or lactate [212]. HSs reduced by metal-reducing bacteria can shuttle electrons and reduce metals with estimated reduction potentials from 0.5 to 0.7 eV [214] and then enhance the formation of the active species of metals, such as the formation of Fe(II) species from Fe(III) oxides [15,17,194].

EESs are particularly relevant to situations when microorganisms have limited access to a critical substrate. For example, an electron acceptor might be poorly soluble (minerals in many groundwater and sedimentary systems), or the substrate might be locally depleted due to rapid consumption by other cells (oxygen in biofilms). On the other hand, the critical substrate might be utilized by another organism in an intimate syntrophic partnership, where the passage of electrons between different microorganisms is required to catalyze a biogeochemical reaction (anaerobic oxidation of methane by a mixed consortium of microorganisms). The latter is carried out by interspecies electron transfer (IET), which is an important mechanism for energy exchange, establishing the basis of cooperative behaviors and community functions in a range of anaerobic microbial communities [215]. As quinone moieties can serve as electron shuttles between the electron-donating and electron-accepting partners, HSs can also shuttle electrons between syntrophic microorganisms [216,217]. In each of these cases, EESs allow the microorganisms at a distance from the terminal electron acceptor to remain metabolically active [18].

Reduced forms of HSs, in turn, can serve as electron donors for anaerobic organisms growing on terminal electron acceptors, such as nitrate [190]. In this case, microorganisms utilize the reduced HSs as a source of energy and utilize other available substrates (acetate, ethanol, fumarate, lactate) as a source of carbon. This adaptation mechanism provides humic-oxidizing microorganisms with a competitive advantage over other heterotrophs in the environment that utilize readily degradable organic compounds as the source of both carbon and energy, which requires greater concentrations for growth [190].

Many microorganisms among the HRMs, including species belonging to the *Geobacter*, *Geothrix*, and *Shewanella* genera, were demonstrated to be capable of coupled oxidation of reduced HSs with nitrate reduction [125,218]. Analyses of humic-oxidizing microorganisms derived from soils and sediments revealed that all isolates were members of the Proteobacteria, mainly nonfermenting facultative anaerobes, demonstrating that this metabolism is widespread throughout the phylum [190,218]. Interestingly, some denitrifiers such as *Paracoccus denitrificans*, which do not belong to HRMs, also use reduced HSs as an electron donor for denitrification [125]. The estimated humic-oxidizing population in the soils and aquifer sediments ranged from 2.3 × 10^1^ to 9 × 10^6^ cells g^−1^ [190,218].

Reduced HSs may serve as an electron donor in anaerobic microbial respiration and can transfer electrons to a variety of organic and inorganic pollutants, thus determining their speciation and degradation [196,219]. Electron transfer to HSs in anoxic systems is considered to suppress the reduction of other terminal electron acceptors, including CO_2_ under methanogenic conditions [189]. Therefore, management of redox processes in the microorganisms–HSs–pollutants system is a promising direction for the development of green biotechnologies for cleaning polluted environments and controlling methanogenesis.

## 6. Nature-Like Bioremediation Technologies Based on HS–Microorganism Interactions

Nowadays, there is a growing interest in developing nature-like technologies, i.e., technologies free of toxic chemicals imitating natural self-purification processes [220]. The considered processes of HS utilization by microorganisms allow us to highlight the following aspects of these interactions, which can be used for the development of nature-like bioremediation technologies:

- The enzymes released by microorganisms to utilize HSs can catalyze oxidative binding of phenols and anilines; this approach can be applied as an alternative to the extraction of pollutants using organic solvents.
- Degradation of HSs by microorganisms can lead to the formation of low-molecular-weight compounds with high bioavailability and, as a result, biostimulating activity; this is a way to utilize low-rank coal or organic wastes to substitute traditional coal liquefaction requiring multistep treatment with chemicals.
- HSs are universal adaptogens that allow microorganisms to survive at high concentrations of toxicants; the mitigating activity of HSs can be used to increase the efficiency of bio-preparation for remediation of polluted environments.
- Participation of HSs in redox reactions can be accompanied by the transformation of organic and inorganic pollutants; degradation of chlorinated organic pollutants may be enhanced under anoxic conditions, and the reduction of some toxic metals followed by lowering their toxicity and mobility can be reached.
- Transfer of electrons from anaerobic respiration through HSs to oxygen may competitively suppress electron transfer to CO_2_, reducing the formation of CH_4_ in temporarily anoxic systems; managing methane emissions is a crucial point both for biogas production and landfill restoration.

The most promising effects of microorganism–HS interactions that can become the basis for the development of nature-like biotechnologies are summarized in Table 5.

The use of the redox activity of HSs seems to be the most promising direction for the application of HSs in water clean-up. The usage of HSs or their quinonoid analogs as redox mediators is recognized as an effective strategy to enhance the biotransformation of toxicants including both organic compounds [26,29,197,243] and inorganic species [29,243]. Extensive research has been conducted to explore the catalytic effects of different electron shuttles on redox biotransformation, mainly for groundwater and wastewater treatment [244]. HSs can be reversibly oxidized and reduced, thereby conferring the capability of serving as electron carriers and accelerating multiple redox reactions [243]. HSs can be reduced by microorganisms in the presence of electron donors commonly found in anaerobic environments (sulfide, ferrous iron, or nitrate) [15,86,185,192]. The reduced HSs can transfer electrons to strong electron acceptors, such as halogenated compounds or nitroaromatics, facilitating their biodegradation [206,226,243].

### 6.1. HS-Facilitated Biodegradation of Organic Contaminants in Soil and Sediments

Collins and Picardal [202] demonstrated that soil organic matter facilitated the dechlorination of CCl_4_ to CHCl_3_ by *Shewanella putrefaciens*, and the observed effect of the enhanced reductive transformation of CCl_4_ was more pronounced in the case of the presence of HAs as compared to FAs. Functional group analysis showed that FAs were characterized by a higher total and carboxylic acidity as compared to HAs; however, both fractions contained similar amounts of total carbonyl groups and quinone carbonyls. The latter indicated the role of functional groups in HSs in determining their redox activity. Similar results were reported by Cervantes et al. [90], who demonstrated that HSs stimulated the reduction of CCl_4_ by *Geobacter* sp., which did not convert CCl_4_ in the absence of HSs.

The positive effects of HSs on the biodegradation of nitroaromatics by *Clostridium* sp. were shown by Bhushan and coauthors [206]. They observed the catalytic effects of coal HAs on the biodegradation of hexahydro-1,3,5-trinitro-1,3,5-triazine (RDX) and octahydro-1,3,5,7-tetranitro-1,3,5,7-tetrazocine (HMX). Along with the increased biotransformation rates of RDX and HMX in the presence of HSs, the extent of mineralization of both pollutants also increased. The removal of RDX was completed in 5 days when HSs were added to the media, while removal in an unamended environment required 10 days. Kwon and Finneran [199] also reported an increased reduction rate of RDX by *Geobacter metallireducens* in the presence of HS analog AQDS compared to unamended controls. The addition of AQDS to Fe(III)-containing microbial cultures resulted in a 5-fold increase in the reduction rate of RDX. Along with chlorinated compounds, other organic pollutants for which the positive role of HSs in anaerobic biodegradation has been shown include azo dye [27,35], benzene [222], hexadecane [225], tetrabromobisphenol A [28], and toluene [26]. Although many of them can easily be decomposed by microorganisms in the presence of oxygen, their degradation is slow under reducing conditions. That is why the development of technologies to accelerate biodegradation in oxygen-free conditions is promising.

Denitrification of ammonium is another area where HSs can be used in the treatment of polluted waters. Wastewaters often need to be treated before discharge because of the high concentrations of ammonium, which can produce high levels of danger and harm to the environment [221]. Microbial denitrification is the main pathway for nitrogen removal both from natural water sources [224] and wastewater [221]. The positive effects of HSs on this process were recently reported [221]. The authors revealed substantial changes in the activated sludge community structure and the dominance of Betaproteobacteria *Thauera* after long-term exposure to coal HAs. Bacteria could utilize HSs as electron shuttles to improve denitrification performance, especially for nitrite reduction. However, the enhanced rate of denitrification was due not only to the ability of HSs to act as an electron carrier; HSs were also found to significantly upregulate the gene expressions and catalytic activities of the key enzymes related to denitrification and the electron transport system’s activity, which accelerated nitrogen oxide reduction. Similar results were reported earlier for HS-mediated denitrification in natural waters by [224]. The authors demonstrated that coal HAs increased the activities of key denitrifying enzymes, including nitrate reductase, nitrite reductase, nitric oxide reductase, and nitrous oxide reductase, thus enhancing the reduction of nitrate and transformation of its intermediates, especially nitrite and nitrous oxide.

Microbial reduction of nitrate may be coupled with anaerobic oxidation of methane (AOM) and plays a crucial role in mitigating methane emissions [187,245]. AOM accompanied by the reduction of HSs, where humic substances serve as terminal electron acceptors, was recently demonstrated for nitrate-reducing anaerobic methanotrophic archaea, subclaster 2d (ANME-2d), which are mainly distributed in paddy soils and freshwater sediments [187]. Valenzuela and coauthors reported that an unclassified genus of the marine benthic group D (MBG-D) family, a proposed microbial player in metal-dependent AOM, also performs AOM coupled with the reduction of HSs and AQDS [246]. Therefore, the mitigation of methane emissions using HSs can be a promising method for landfill treatment when the CH_4_ concentration in the landfill gases is reduced to less than 20% after the active phase of MSW degradation and the thermal technologies are less likely to be serviceable [247].

Mitigation of methane emissions by HSs is thought mainly to be caused by stimulating AOM [187,245], but inhibition of methanogens’ growth should also be considered [217,248]. Increasing HS concentrations were demonstrated to inhibit the growth of hydrogenotrophic methanogens from the genus *Methanobacterium* and syntrophic bacteria from the genus *Syntrophomonas*, which resulted in a decrease in methane production [217]. Methanogenesis involves various biological processes requiring complex syntrophic microbial communities, and the overall methanogenesis rate is typically limited by hydrolysis [249]. Therefore, other proposed mechanisms of methane emissions mitigation by HSs include HSs binding active sites of relevant hydrolytic enzymes, thereby preventing access to substrates [233], or HSs binding hydrolytic bacterial cell walls, disrupting cell membrane integrity and/or essential cellular transport processes [250].

### 6.2. Reduction of Metals by HSs

The ability of HSs to reduce a number of transition metals is also of considerable interest from a water treatment point of view. These toxicants may exist in several oxidation states, and microorganisms may reduce a wide range of them, causing either a decrease or an increase in mobility [219]. The reduction of soluble Cr(VI) to sparingly soluble Cr(III), for example, results in decreased mobility of chromium in the environment. Therefore, the reduction of Cr(VI) to Cr(III) is taken as an effective remediation strategy for Cr(VI)-contaminated sites [29,30,31]. Therefore, the promotion of Cr(VI) bioreduction using HSs can be a novel approach to remove Cr(VI) from Cr(VI)-contaminated sites as an alternative to the existing remediation techniques, such as chemical reducing agents, ion exchange, electrochemical techniques, and others [31].

In contrast to the Cr(VI) to Cr(III) reduction process, the transition of Fe(III) to Fe(II) results in an increase in iron mobility. Anaerobic microbial biosolubilization of iron is called bioleaching. It is an attractive approach for iron extraction from recalcitrant ores and reprocessing waste materials from mining operations with remarkable environmental benefits [241]. HS-mediated anaerobic microbial bioleaching is an advantageous alternative to metal solubilization using chemical leaching processes with strong acids, which are favorable only when there are high levels of metals in wastes [241]. Reduction of Fe(III) to Fe(II) in the presence of HRMs and HSs was demonstrated for jarosite [241] and goethite [186], which are mined for iron as well as for ferrihydrite, which is a precursor of goethite [240].

An example of a possible application of HSs in clean-up technologies based on metal oxidation is gas demercurization. Elemental mercury Hg(0) can compose up to 94% of the total Hg in coal-fired flue gas. Elemental mercury is highly volatile, water-insoluble, and difficult to remove. Techniques for the demercuration of flue gas primarily focus on Hg(0) oxidation to mercury Hg(II), followed by removing it using existing air pollution control devices such as wet flue gas desulfurization systems, electrostatic precipitators, and fabric filters [251]. However, Hg(II) is more toxic, chemically reactive, and highly bioavailable for methylation. All of this together may cause the problem of environmental pollution. The paper [183] considered the possibility of bioconversion of Hg(0) in a sulfate-reducing membrane biofilm reactor (MBfR). The MBfR achieved effective Hg(0) removal by sulfate bioreduction coupled with Hg(0) oxidation followed by HgS formation. Though the role of HSs in the redox state of mercury was not studied, the authors demonstrated strong Hg(II) complexation by the functional groups −SH, −OH, −NH−, and −COO− in HSs from extracellular polymeric substances produced in the biofilm. Hg in the complexes with HSs may further react with biogenic sulfides to form HgS [183]. Another study found that this complexation may also occur by means of ligand-induced oxidative complexation due to the strong tendency of Hg(0) to react with reduced sulfur or thiols in the HSs [252].

The redox activity of HSs could be used to increase power production in a microbial fuel cell (MFC), which is a device that directly converts microbial metabolism into electricity using electrochemical processes [242]. Coal HAs were used as exogenous electron shuttles to the MFC based on xylose as the electron donor and microorganisms from domestic wastewater. At 0.5 g/L HA addition, the maximum power density increased by 45%. Additionally, the comparatively higher power generation under the addition of HAs was sustained for a longer period than that without the addition of HAs [242].

Reductive biodegradation mediated by HSs can arguably be effective not only in water but also in solid substrates where anoxic conditions can be found, such as paddy soils [228]. Instead, most studies showing facilitated biodegradation of organic pollutants after HS amendment explained the observed effect either by mitigating the activity of HSs on bacteria in the presence of a toxicant [85,230,231] or by increasing its bioavailability [19,21,51]. Another promising area for HS application to reduce soil contamination is an enzymatic transformation and covalent binding of organic pollutants to HSs (oxidative coupling). As a result of covalent binding, xenobiotics become integral parts of HSs. As such, organic pollutants are resistant to release by microbial activity or chemical treatment. Therefore, covalent binding appears to be the only immobilization process that may be considered environmentally desirable [182]. Among compounds that may undergo covalent binding to HSs are phenols and aromatic amines ([182] and citations therein, [227]). Enzymes catalyzing oxidative coupling are LMEs, which can oxidize phenolic compounds via creating phenoxy and carboxy radicals and can oxidize nonphenolic compounds via cation radicals [10]. LMEs are enzymes related to HS degradation in the environment. Therefore, the application of LMEs or microorganisms actively degrading HSs might provide a promising approach for nature-inspired technologies.

### 6.3. Biosolubilization for Lignite Utilization

Degradation of HS-containing organic solid deposits may be required when disposing of lignites. The utilization of lignite poses a serious threat to the environment. Coal use for electricity production brings about substantial emissions of harmful gases such as sulfur and nitrogen oxides [234]. Biosolubilization of lignite might yield liquid products, which can be processed into utilizable energy [22,253]. A variety of aromatic and aliphatic compounds, which can be found in biosolubilized lignite, could serve as chemical feedstock for subsequent processes such as methanogenesis [22,236]. They can be also used as raw material for the production of valuable chemicals [163]. Biosolubilization of lignite has substantial advantages over thermochemical conversion. These include (1) operation at atmospheric temperature and pressure; (2) conversion of a substrate into a single-phase product without a large quantity of byproducts; and (3) microorganisms, which obtain hydrogen from water, not requiring an external hydrogen source for lignite solubilization [254].

In addition, biosolubilization is a promising technology for converting low-rank coal into value-added products [237,254]. For example, accumulation of polyhydroxyalkanoic acids (PHAs) to levels reaching up to 7–8% of the cell dry mass was observed in [237] during biosolubilization by the bacteria *Pseudomonas oleovorans* and *Rhodococcus ruber*. PHAs are biodegradable microbially synthesized polymers, which could be considered as alternatives to the conventional petrochemical plastics. PHAs are deposited in the cell as water-insoluble intracellular granules, which can be easily isolated from lyophilized cells [237].

In 1981, hard coal was used for the first time as the sole source of carbon and energy for microorganisms, and its partial dissolution was observed [254]. Later on, total dissolution of lignite by two fungal species, *Polyporus versicolor* and *Poria monticolar*, was demonstrated [255]. Bioproducts from lignite, obtained by fungal biodegradation, had a higher content of nitrogen and lower molecular mass as compared to undegraded HSs. They were capable of stimulating biological activity in soil [235,256]. High N content in biotransformed HAs could have a potential application in agriculture, as N is essential for plant growth [54,256].

Along with fungal biodegradation, microbial biosolubilization of coal was reported. Strains of *Acinetobacter*, *Bacillus*, *Enterobacter*, *Escherichia*, *Microbacterium*, *Pseudomonas*, *Rhodococcus*, *Staphylococcus*, and *Streptomyces* were the most active in coal degradation [20,234,237,257,258]. Quite a few patents on coal biosolubilization can be found, which mostly rely on the use of fungal LMEs [259,260,261] or the bacteria *Streptomyces* sp. [262]. However, an efficient and economically viable coal biosolubilization process is yet to be developed. The main reason for this is the difficulty in handling coal in bioreactors and the loss of process stability due to the complex regulation of the solubilization mechanisms [258] and low conversion rates [20,263].

Another important reason is the impossibility of complete degradation of HSs by only one type of bacterium [100]. It has been shown that in nature, this process is implemented by a succession of complex microbial communities that replace each other depending on the stage of biodegradation [6,100,106,264]. A striking example of the work of such communities is the intestinal tract of soil-feeding species of termites that utilize substrata and HSs. Their ability to degrade HSs relies on their partnership with a diverse community of bacterial, archaeal, and eukaryotic gut symbionts. The high efficiency of their minute intestinal bioreactors makes termites a promising model for the industrial conversion of lignocellulose and HSs into microbial products and the production of biofuels [265].

## 7. Research Needs

Despite a substantial amount of data indicating the prospects of using HSs for green technologies based on the interactions of humic substances with microorganisms (Table 5), ready-to-use technologies are still lacking. First of all, this is due to the general problems of using HSs identified in the recent paper by Olk and coauthors [32]. The authors formulated four main reasons that prevent the widespread use of humic materials in agriculture, namely an insufficient number of field studies addressing the effects on humic product efficacy depending on environmental and management factors, a need for a mechanistic explanation of HS activity, a lack of quality control of humic products, and an insufficient number of long-term field trials.

**Figure 1:**
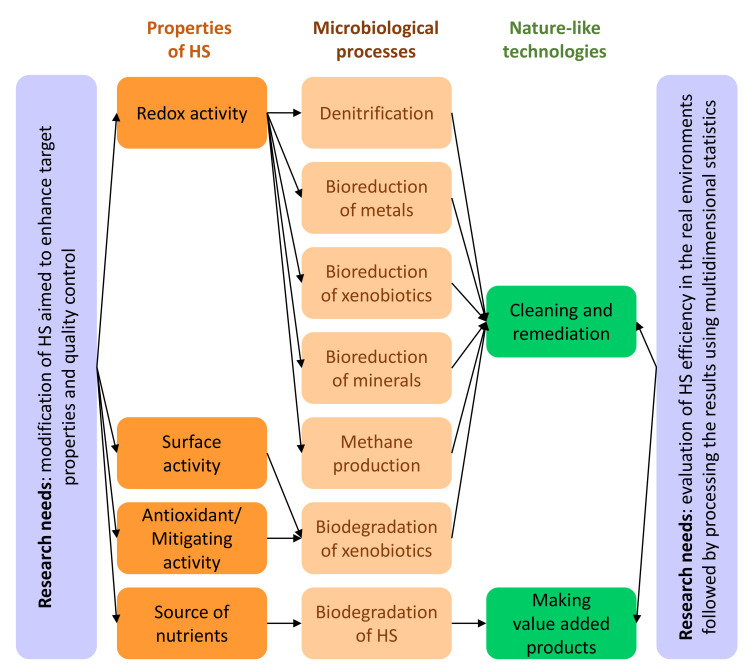
Principal research needs for microorganism–HS interactions that can support the development of nature-like technologies.

With the exception of understanding the mechanism of action of HSs, the other listed reasons are also relevant for the development of green technologies based on HS–microorganism interactions (Figure 1).

A lack of quality control of humic products is of special importance as the internal variability observed among humic materials and their fractions, mainly redox properties, can change their properties entirely [78,228,232,243]. Reducible moieties in HSs were revealed to cover a wide range of apparent standard reduction potentials at pH 7 from +0.15 to −0.3 V [92]. The electron-carrying capacities (ECCs) of eight HSs measured by repeating sequential reduction and oxidation steps ranged from 25 to 538 μequiv e^−^ g^−1^, depending on the pH and the reduction catalytic system [91]. The electron-donating capacities (EDCs) of 15 HSs and natural organic matter (NOM) at applied redox potentials Eh = 0.61 V and pH 7 ranged from 0.47 to 2.65 mmol e^−^ g^−1^ (77). In general, the aquatic HSs had lower ECC and higher EDC values than the terrestrial HSs [77,91]. Such a wide variability of redox properties of HSs resulted in the irregularity of the observed effects.

Tan and coauthors studied the effects of HS additions on CH_4_ production under anoxic conditions using three standards of humic material produced by the International Humic Substances Society. They were Elliot soil humic acid (ESHA), Pahokee peat humic acid (PPHA), and Suwannee river humic acid (SRHA) [232]. ESHA, PPHA, and SRHA exerted different intensities of CH_4_ production suppression; the order of suppression intensities was the same as that of the electron-accepting capacities of HSs.

The dependence of pentachlorophenol (PCP) biodegradation on peculiar properties of HSs under reducing conditions was established by [226]. The hydrophobicity and molecular weight of HSs were demonstrated as the main properties determining the efficiency of PCP bio-dechlorination [226]. In another study, the effect of HAs extracted from forest, paddy, and peat soil on the microbial community involved in anaerobic mineralization of PCP was estimated [228]. The results show that the effect of biotransformation processes of PCP depended on the HAs used; the prominent microorganisms for the mineralization also varied. The genera *Methanosarcina* and OP11 *incertae genera* were prominent after treatment with forest HAs, whereas *Burkholderia* and *Methanobacterium* were prominent when paddy or peat soil HAs were added [228]. The latter shows that the efficiency of HSs in the processes of reducing biodegradation can be influenced not only by their redox properties but also by their ability to influence the composition of the microbial community.

Along with redox activity and the ability to stimulate the development of certain microorganisms, environmental conditions are also of great importance. Li and coauthors studied the influence of Cu(II) on the efficiency of water treatment with HSs [266]. The authors selected copper as a well-known heavy metal deteriorating biological wastewater treatment processes. It was found that although the addition of HSs resulted in a slight increase in nitrogen removal rate, copper or its combination with HSs had the opposite result [266].

Yuan and coauthors revealed a strong dependence of bio-dechlorination of PCP in the presence of different HSs on Fe_2_O_3_ or Fe_3_O_4_ reduction environments [226]. Under Fe_2_O_3_ reduction conditions, relatively hydrophilic and high-molecular-weight HSs were more efficient for PCP biodegradation. In contrast, the hydrophobic and low-molecular-weight components were the main functional components for PCP bio-dechlorination in Fe_3_O_4_ reduction environment [226]. The authors concluded that the effective components within HSs for dechlorination of PCP would be changed with the type of Fe(III) mineral in the environment.

Thus, existing research allows us to consider at least three factors that determine the effectiveness of HSs: (1) the redox activity of HSs, (2) the ability of HSs to alter the composition of the microbial community, and (3) environmental conditions.

To overcome the problem of low redox activity of HSs, two different strategies to synthesize quinoid-enriched humic materials with enhanced redox properties were developed [267]. The first approach was related to the oxidation of phenolic fractions associated with the humic aromatic core. In a second strategy, polycondensation of these phenolic fragments was carried out with hydroquinone and catechol. Redox characterization of the copolymers obtained revealed that the reducing capacity of this synthesized humic material was much higher than that of the parent materials and the oxidized derivatives. Therefore, preferential application of the co-polycondensation approach was advised. Considering the wide variety of redox reactions in which HSs could be useful, further research is required in order to obtain engineered HSs that have desired redox characteristics [243]. However, the success of the approach associated with the use of modified humic substances was demonstrated using sulfonated leonardite HAs to increase reductive decolorization of azo dye Reactive Red 2 and dechlorination of CCl_4_ [27].

Pursuing the same goal, Wei and coauthors compared the effects of 18 HAs from the mesophilic, thermophilic, and mature phases of protein-, lignocellulose-, and lignin-rich composting on catalyzing the bioreduction of Fe(III)–citrate by *Shewanella oneidensis* MR-1 in temporarily anoxic laboratory systems. They showed the Has from lignocellulose- and lignin-rich composting, especially in the thermophilic phase, significantly promoted the bioreduction of Fe(III), and Has from protein-rich materials significantly suppressed Fe(II) production [78].

Evaluation of the efficiency of HSs, in turn, should take into account their influence on the composition of microorganisms and environmental features. This is possible only by conducting mass experiments using consortia of microorganisms and samples of real polluted environments. These studies should be accompanied by a detailed study of successions of the microbiological community and a detailed description of polluted environments, followed by processing the results using multidimensional statistics. This will allow us to establish the limits of applicability of humic materials and develop cleaning technologies based on humic substances.

## Figures and Tables

**Table 1:** Some biologically active compounds found in HSs.

Biologically Active Compounds	HSs	Content, %	Ref.
Amino acids ^1^			
A sum of Ala, Arg, Asp, Cys, Glu, Gly, His, Ile, Leu, Lys, Met, Phe, Pro, Ser, Thr, Tyr, Val	Soil HAs	6–17	[61]
	Soil HAs	9–16	[62]
	Soil HAs	6–8	[63]
	Peat HAs	3–7	
	Soil HAs	9	[5]
	Soil FAs	7	
	Riverine FAs	3	
	Riverine HAs	6	
	Marine FAs	4	
Carbohydrates			
A sum of fructose, galactose, glucose, mannose, rhamnose, and xylose	Soil FAs	4	[5]
	Soil HAs	10	
	Riverine FAs	0.1	
	Riverine HAs	0.1	
	Marine FAs	1	
A sum of glucose, galactose, mannose, xylose, arabinose, fucose, and rhamnose	Soil HAs	3–9	[64]
	Soil FAs	3	
A sum of hexose, pentose, and uronic acid	Soil FAs	4–8	[65]
Lipids			
Fatty acids	Soil HMA	5–10	[66]
	Soil HAs	41–375 nmol/g	[67]
	Soil HAs	0.1–10	[68]
	Soil FAs	0.1–9	
Aromatic acid saponification byproducts	Peat HAs	2 × 10^−3^	[69]
	Peat FAs	9 × 10^−4^	
Plant hormones			
Gibberellin-like substances	Soil HAs	≥1 × 10^−5^	[70]
Indole-3-acetic acid	Vermicompost HAs	0.33	[71]
	Soil HAs	0.01–0.05	[72]

^1^ Amino acids: Ala—alanine; Arg—arginine; Asp—aspartic acid; Cys—cysteine; Gln—glutamine; Glu—glutamic acid; Gly—glycine; His—histidine; Ile—isoleucine; Leu—leucine; Lys—lysine; Met—methionine; Phe—phenylalanine; Pro—proline; Ser—serine; Thr—threonine; Tyr—tyrosine; Val—valine.

**Table 2:** Some genera of humic-degrading bacteria.

Genus	HSs	Ref.
**Phylum *Proteobacteria*** (**Gram-Negative**)		
Class *Alphaproteobacteria*		
*Agrobacterium*	Soil HAs	[101]
*Aquaspirillum, Erythrobacter*	Aquatic HAs from estuarine water	[100]
*Ahrensia*, *Erythrobacter*, *Oceanibulbus*, *Roseovarius*, *Sphingobium*, *Sphingopyxis*, *Sulfitobacter*, *Thalassospira*	Aquatic HAs from freshwater stream in a peat bog	[6,107]
*Aminobacter*, *Ochrobactrum*, *Sphingopyxis*	Coal HAs	[107]
Class *Betaproteobacteria*		
*Acidovorax*, *Herbaspirillum*, *Methylophilus*, *Polynucleobacter*	Aquatic HSs from a humic lake	[106]
*Delftia*	Coal HAs	[107]
*Variovorax*	Soil HAs	[108]
Class *Gammaproteobacteria*		
*Acinetobacter*, *Aeromonas*, *Buttiauxella*	Coal HAs	[107]
*Alteromonas*	Aquatic HAs from freshwater stream in a peat bog	[6]
*Pseudomonas*	Soil HAs	[12]
	Soil HAs	[101]
	Aquatic HSs from a humic lake	[104]
	Soil HAs and FAs	[103]
	Lignite HAs	[109]
	Soil HAs	[108]
	HSs from *Quercus rubra*, *Hamamelis virginiana*, and *Zea mays* leaves	[110]
	Coal HAs	[111]
	Coal HAs	[107]
**Phylum *Bacteroidetes*** (**Gram-Negative**)		
Class *Bacteroidetes*		
*Bacteroides*	HSs from landfill leachate	[2]
Class *Flavobacteriia*		
*Chryseobacterium*	Coal HAs	[107]
**Phylum *Firmicutes*** (**Gram-Positive**)		
Class *Bacilli*		
*Bacillus*	Soil HAs	[101]
	Soil HAs and FAs	[103]
	HSs from landfill leachate	[2]
	Aquatic HAs from estuarine water	[100]
	Leonardite HAs	[20]
	Coal HAs	[107]
	Soil HAs	[112]
*Paenibacillus*	Aquatic HAs from estuarine water	[100]
	HSs from landfill leachate	[2]
	Coal HAs	[107]
*Staphylococcus*	HSs from landfill leachate	[2]
Class *Clostridia*		
*Clostridium*	Coal HAs and HAs from diatomite layer	[105]
**Phylum *Actinobacteria*** (**Gram-Positive**)		
Class *Actinobacteria*		
*Arthrobacter*	Soil HAs	[101,107]
*Agromyces*, *Kocuria*, *Nocardioides*	Coal HAs	[107]
*Dactylosporangium*, *Micromonospora*, *Microtetraspora*, *Nocardia*, *Streptosporangium*, *Thermomonospora*	Soil HAs	[113]
*Microbacterium*	Soil HAs	[112]
*Streptomyces*	Manure and soil HAs	[114]
	Soil HAs	[113]
	Soil HAs and FAs	[103]
	Soil HAs	[115]
	Soil HAs	[116]
	Soil HAs	[117]
	Coal HAs	[107]

**Table 3:** Some genera of HS-degrading fungi.

Genus	HSs	Ref.
**Phylum *Ascomycota***		
Class *Dothideomycetes*		
*Alternaria*	Soil HAs and FAs	[147]
	Leonardite HAs	[148]
*Cladosporium*	Aquatic HAs from a bog lake	[149]
	Leonardite HAs	[148]
	Riverine HAs	[150]
*Phoma*	Soil HAs and FAs	[147]
	Leonardite HAs	[148]
Class *Eurotiomycetes*		
*Aspergillus*	Manure and soil HAs	[114]
	Soil HAs	[151]
*Paecilomyces*	Coal HAs	[152]
	Soil HAs and FAs	[153]
	Soil HAs and FAs	[147]
*Penicillium*	Soil HAs	[101]
	Manure and soil HAs	[114]
	Soil HAs	[151]
	Coal HAs	[154]
Class *Leotiomycetes*		
*Geomyces*	Leonardite HAs	[148]
Class *Sordariomycetes*		
*Chalara*	HAs from *Picea abies*	[155]
*Clonostachys*	Soil HAs and FAs	[147]
*Fusarium*	Manure and soil HAs	[114]
	Leonardite HAs	[148]
*Trichoderma*	Coal HAs	[154]
**Phylum *Basidiomycota***		
Class *Agaricomycetes*		
*Bjerkandera*	Soil HAs	[156]
	Coal HAs	[157]
*Clitocybula*	Soil HAs	[156]
*Gymnopilus*	Soil HAs	[156]
*Hypholoma* (*Naematoloma*)	Coal HAs	[158]
	Soil HAs	[156]
	Synthetic HAs	[159]
*Kuehneromyces*	Soil HAs	[156]
*Lenzites*	Soil HAs	[160]
*Phanerochaete*	Soil HAs	[161]
	Soil HAs and FAs	[116]
	Coal HAs	[162]
	Coal HAs	[152]
	Lignite HAs	[163]
	HAs from biosolids compost	[164]
*Pleurotus*	Coal HAs	[152]
	Soil HAs	[156]
*Polyporus*	Aquatic HAs from a bog lake	[149]
	Coal HAs	[165]
*Pycnoporus*	Coal HAs	[165]
*Trametes* (*Coriolus*)	Soil HAs and FAs	[116]
	Coal HAs	[152]
	Soil HAs	[160]
	Leonardite HAs, peat HAs, HAs from biosolids compost	[164]
	Coal HAs	[84]
*Stropharia*	Soil HAs	[156]
Class *Basidiomycetes*		
*Collybia*	Soil-litter and litter HAs	[166]

**Table 4:** Some genera of humic-reducing microorganisms.

Genus	HSs	Ref.
**Phylum *Proteobacteria*** (**Gram-Negative**)		
Class *Alphaproteobacteria*		
*Brevundimonas*, *Devosia*, *Phyllobacterium*, *Rhodobacter*	Compost HAs	[196]
Class *Betaproteobacteria*		
*Comamonas*	Model HA (AQDS)	[197]
	Compost HAs	[196]
*Pusillimonas*, *Rubrivivax*, *Janthinobacterium*	Compost HAs	[196]
Class *Deltaproteobacteria*		
*Desulfobacca*	Compost HAs	[196]
*Geobacter*	Soil HAs	[16]
	Soil HAs and Model HA (AQDS)	[90]
	Riverine, soil, peat, and coal HAs	[198]
	Model HA (AQDS)	[199]
	Soil, leonardite, and compost HAs	[88]
	Model HA (AQDS)	[200]
Class *Gammaproteobacteria*		
*Acinetobacter*, *Psychrobacter*, *Pseudomonas*, *Pseudoxanthomonas*, *Pantoea*	Compost HAs	[196]
*Aeromonas*	Model HAs (AQC, AQS, AQDS, 2-HNQ, 5-HNQ)	[201]
*Shewanella*	Model HAs (AQDS, AQS)	[35]
	Riverine, soil, peat, and coal HAs	[198]
	Soil HAs and FAs and humin	[202]
	Peat, riverine, soil, and leonardite HAs	[189]
	Model HAs (AQC, AQS, AQDS, 2-HNQ, 5-HNQ)	[201]
	Peat HAs	[203]
	Soil DOM	[204]
	Compost HAs	[78]
*Sideroxydans*	Peat HAs	[205]
**Phylum *Bacteroidetes*** (**Gram-Negative**)		
Class *Sphingobacteriia*		
*Sphingobacterium*	Compost HAs	[196]
**Phylum *Firmicutes*** (**Gram-Positive**)		
Class *Bacilli*		
*Bacillus*	Soil HAs, HAs from midgut, hindgut, and feces of *Pachnoda ephippiata*	[143]
	Model HAs (AQDS, AQS)	[29]
	Compost HAs	[196]
*Paenibacillus*, *Lysinibacillus*, *Sprorosarcina*, *Ureibacillus*, *Facklamia*	Compost HAs	[196]
Class *Clostridia*		
*Clostridium*	Coal HAs	[206]
*Desulfitobacterium*	Coal HAs, model HA (AQDS)	[192]
*Sedimentibacter*, *Tissierella*, *Proteiniborus*, *Coprococcus*	Compost HAs	[196]
**Phylum *Actinobacteria*** (**Gram-Positive**)		
Class *Actinobacteria*		
*Kocuria*	Model HA (AQDS)	[207]
*Corynebacterium*	Model HA (AQDS)	[208]
	Coal FAs and HAs, model HAs (AQDS, AQS, AQC),	[186]
*Arthrobacter*, *Corynebacterium*, *Dietzia*, *Leucobacter*	Compost HAs	[196]
**Phylum *Deinococcus-Thermus*** (**Gram-Positive**)		
Class *Deinococci*		
*Deinococcus radiodurans*	Model HA (AQDS)	[209]

AQDS—anthraquinone-2,6-disulfonate; AQS—anthraquinone-2-sulfonate; AQC—9,10-anthraquinone-2-carboxylic acid; 2-HNQ—2-hydroxy-1,4-naphthoquinone; 5-HNQ—5-hydroxy-1,4-naphthoquinone.

**Table 5:** Some of the microorganism–HS interactions that can support the development of nature-like bioremediation technologies.

Biological Agent	HSs	Effect	Ref.
**Water purification/treatment**			
Consortium of microorganisms from activated sludge	Coal HAs	The dominance of *Thauera* after long-term exposure to HSs resulted in increased denitrification	[221]
Consortium of microorganisms from biofilm	Coal HAs	Enhanced TBBPA biodegradation in the bioelectrochemical system	[28]
Consortium of microorganisms from sludge	Sludge HAs	Increased anaerobic bioreduction of Cr(VI)	[30]
Consortium of microorganisms from sediment	Soil HAs, model HA (AQDS)	Increased toluene biodegradation	[26]
Consortium of microorganisms from soil and sediment	Soil HAs	Increased reductive benzene degradation	[222]
Consortium of microorganisms from soil, sediment, and anaerobic granular sludge	Sulfonated leonardite HAs, soluble or immobilized onto anion exchange resin	Increased reductive decolorization of azo dye Reactive Red 2 and reductive dechlorination of CCl_4_	[27]
*Bacillus* sp. 3C3	Model HAs (AQS, AQDS)	Enhanced Cr(VI) reduction	[29]
*Clostridium* sp. EDB2	Coal HAs, model HA (AQDS)	Enhanced degradation of RDX and HMX	[206]
*Comamonas koreensis* CY01	Model HA (AQDS)	Enhanced reductive dechlorination of 2,4-D	[197]
*Corynebacterium humireducens* MFC-5	Coal HAs and FAs	Biodegradation of 2,4-D	[186]
*Dehalococcoides* spp.	Model HA (AQDS)	Increased reductive dechlorination of C_2_HCl_3_	[223]
*Deinococcus radiodurans* R1	Model HA (AQDS)	Increased reduction of Tc(VII) and U(VI)	[209]
*Paracoccus denitrificans*	Coal FAs	Enhanced denitrification	[224]
*Rhodococcus erythropolis* S67 and X5	Peat HA	Increased utilization of C_16_H_34_	[225]
*Shewanella decolorationis* S12	Model HAs (AQS, AQDS)	Acceleration or inhibition of azoreduction depending HA concentration	[35]
*Sh. oneidensis* MR-1	Compost HAs	Facilitated bio-dechlorination of PCP under Fe(III) reduction conditions	[226]
*Sh. oneidensis* MR-1	Complex goethite-reduced HAs	Enhanced reduction of Cr(VI) to Cr(III)	[31]
*Sh. oneidensis* MR-1	Compost HAs	Enhanced anaerobic transformation of PCP	[184]
*Streptomyces* sp.	Soil HAs	Increased decolorization of water	[117]
**Soil/slurry/sediment remediation/biosolid treatment**			
Consortium of anaerobic microorganisms from cow manure	Soil HAs	Increased transformation and covalent binding of 2,4,6-TNT in the presence of laccase	[227]
Consortium of microorganisms from paddy soil	Soil HAs	Enhanced PCP biodegradation attributed to the quinine groups in HAs that functioned as redox mediators	[228]
Consortium of microorganisms from soil	Lignite HAs	Increased decomposition of PAHs due to increased bioavailability	[19]
Consortium of microorganisms from soil	HAs from mechanically activated peat	Increased biochemical oxidation of oil hydrocarbons	[229]
Consortium of microorganisms from soil	Soil HAs	Increased phenanthrene biodegradation due to increased bioavailability	[21]
Consortium of microorganisms from soil	Soil HAs	Increased or decreased pyrene biomineralization depending on concentration due to increased bioavailability	[51]
Consortium of microorganisms from soil	Coal HAs	Enhanced biodegradation of dibutyl phthalate due to mitigating activity of HSs	[85]
Phenoloxidases	HSs present in soil	Covalent binding of phenols and anilines	[182]
*Pseudomonas aeruginosa*	Soil HAs	Increased biodegradation of DBDE due to mitigating effect of HSs on copper	[230]
*P. azotoformanss* ACP1, *P. aeruginosa* ACP2, *P. putida* ACP3 from soil	Coal HAs	Enhanced decompositions of acephate due to mitigating activity of HSs	[231]
**Methane consumption/production/suppression**			
Consortium of microorganisms from anaerobic granular sludge	FAs from MSW leachate	Decreased CH_4_ production	[217]
Consortium of microorganisms from paddy and wetland soils	Soil, peat, riverine HAs	Suppression of CH_4_ production under anoxic environments	[232]
Consortium of microorganisms from piggery wastewater	Coal HAs	Reduction or increase in CH_4_ production depending on HA concentration and pH	[233]
Nitrate-reducing AOM microorganisms	Coal HAs	Mitigation of CH_4_ emission	[187]
**Value-added product production**			
*Bacillus* sp. Y7	Lignite	HAs with high N/O and C/O ratios	[234]
*Penicillium* sp. P6	Lignite	HSs with high content of FAs	[235]
*Penicillium* sp. P6	Lignite	HAs with high N content	[54]
*Phanerochaete chrysosporium*	Lignite	Solubilized lignite for CH_4_ production	[236]
*Phanerochaete chrysosporium*	Lignite HAs	Raw material for production of valuable chemicals and extending the commercial utilization of coal	[163]
*Pseudomonas oleovorans* and *Rhodococcus ruber*	Lignite	PHAs accumulated in the microbial cells	[237]
*Rhizopus oryzae* AD-1	Subbituminous coal	HAs with high N content	[238]
White-rot fungal strains extracted from decaying woods	Coal HAs	Decolorization and depolymerization of HAs	[239]
Bacterial communities	Leonardite	HAs with plant-hormone-like activity	[20]
Fungal isolate MW1	Lignite	A variety of aromatic and aliphatic compounds, which could serve as chemical feedstock for subsequent processes such as methanogenesis	[22]
**Mining processes**			
*Corynebacterium humireducens* MFC-5	Coal HAs and FAs	Bioreduction of goethite	[186]
*Geobacter metallireducens*	Aquatic HAs and FAs from groundwater	Bioreduction of ferrihydrite	[240]
*Shewanella putrefaciens* and a natural consortium	Model HA (AQDS)	Bioreduction of jarosite/bioleaching/metal recovery	[241]
**Flue gas cleaning/demercuration**			
Mercury-oxidizing/sulfate-reducing bacteria	HSs extracted from biofilm	HgS and HA-Hg are two dominant products of Hg^0^ bio-oxidation	[183]
**Electricity generation**			
Consortium of microorganisms from domestic wastewater	Coal HAs	Increase in power density and Coulombic efficiency	[242]

2,4,6-TNT—2,4,6-trinitrotoluene; 2,4-D—2,4-dichlorophenoxyacetic acid; DBDE—decabromodiphenyl ether; HMX—octahydro-1,3,5,7-tetranitro-1,3,5,7-tetrazocine; PCP—pentachlorophenol; PHAs—polyhydroxyalkanoates; RDX—hexahydro-1,3,5-trinitro-1,3,5-triazine; TBBPA—tetrabromobisphenol A; MSW—municipal solid waste; AOM—anaerobic oxidation of methane.
